# The adult social care outcomes toolkit easy read for older people (ASCOT-ER OP): an exploratory factor analysis and Rasch validation study

**DOI:** 10.1007/s11136-026-04169-0

**Published:** 2026-03-13

**Authors:** Stacey Rand, James Caiels, Rasa Mikelyte, Ann-Marie Towers

**Affiliations:** 1 University of Kent, Canterbury, UK; 2 Kings College London, London, UK

**Keywords:** Quality of life · Social care · Older adults · Outcomes · ASCOT

## Abstract

**Purpose:**

ASCOT easy read for older people (ASCOT-ER OP) is an adaptation of the Adult Social Care Outcomes Toolkit (ASCOT-SCT4) that was co-produced with older adults and their families to improve accessibility and feasibility of completion. This study aimed to examine the structural validity of ASCOT-ER OP using exploratory factor analysis (EFA) and Rasch analysis.

**Methods:**

Data were collected by British Red Cross (BRC) as part of short-term support for adults living at home, post-crisis or hospital discharge (‘Support at Home’). ASCOT-ER OP was collected at initial assessment (baseline) and at the end of support (follow-up), typically no longer than 12 weeks later. Only follow-up data were analysed in this study. EFA was conducted to assess structural validity against the single factor structure of ASCOT-SCT4. Overall fit to the Rasch model was examined alongside assessment of unidimensionality, local independence, item fit, response threshold ordering and differential item functioning (DIF) by age group (18–64 and 65 + years).

**Results:**

ASCOT-ER OP had a single factor structure in EFA. There was good fit to the Rasch model without significant breach of assumptions. Item fit was satisfactory. There was no evidence of disordered thresholds, but suboptimal distinguishability between response categories at some thresholds. These aligned to instances of < 10 ratings per category and/or were consistent with the properties of the ASCOT-SCT4. There was evidence of DIF by age group for two items: *Personal Safety* and *Home comfort and cleanliness*.

**Conclusions:**

EFA and Rasch support the structural validity of ASCOT-ER OP as a unidimensional measure.

**Supplementary Information:**

The online version contains supplementary material available at 10.1007/s11136-026-04169-0.

## Background

The Adult Social Care Outcomes Toolkit (ASCOT) is a suite of measures designed for use in social care settings. The original measure (ASCOT-SCT4) was developed as a questionnaire for self-report or interviewer-assisted completion by adults with different care needs [1]. Whilst there are other QoL measures suitable for use with adults accessing social care, such as ICECAP-O [2], ASCOT-SCT4 specifically measures social care-related quality of life (SCRQoL), i.e., the quality of life (QoL) attributes affected by social care services and valued by people accessing services. It is more sensitive as a measure of the outcomes of social care services [3, 4] and is especially suited to the evaluation of social care policy and interventions [5]. Psychometric studies of ASCOT-SCT4 have been conducted with samples of adults accessing care services in England, aged 18–64 years and over 65s, across care settings [6–9]. ASCOT measures have been applied in social care research [5], care planning and assessment [10], improving quality and effectiveness of care services [11, 12] or understanding long-term care system performance [13].

An important consideration in collecting and applying QoL outcomes data in social care, like ASCOT-SCT4, ICECAP-O or other multi-item QoL measures that capture complex constructs, is how to support inclusion. Systematic exclusion in data collections used to inform policy and guide decision-making may introduce bias; it is also ethically problematic based on equity and human rights [14, 15]. This is especially important in data collections with people accessing social care services, who may have needs that affect their ability to understand and respond to standard questionnaires. Previous work with ASCOT measures has explored the potential of different formats [16], methods [12] and modes of data collection [17] to support inclusion by facilitating comprehension, recall, weighing up options, and response.

Inclusion has become increasingly important given UK and international population trends. More older people live in traditional housing, with fewer in care homes [18]. Many live alone, and the likelihood of living alone increases with age, although trends vary globally [19, 20]. The number of older people without children or close family is also rising, especially in the Global North [21, 22], which is expected to affect demand for care services [23]. Globally, the number of people living with dementia is also increasing, although prevalence and incidence rates vary by country [24]. The amount of time people spend living with major illness toward the end of life is projected to increase [25]. These trends are highly relevant to adult social care provision: for example, in the UK, an estimated 60% of people receiving homecare and 70% of those in care homes have dementia [26]. The increasing complexity and severity of needs of people supported by adult social care, such as dementia, may unsurprisingly make it more difficult for people to report QoL using standard self-report tools. Proxy report can offer a pragmatic way to collect data that considers a person’s perspective in the absence of self-report; however, it does not replace or equate to self-report [15]. Therefore, it is ideal, wherever possible, to collect people’s views of their QoL, directly, which is why adapted methods are important and vital role in enabling wider inclusion in data collections.

This principle was behind development of the ASCOT easy read for older people (ASCOT-ER OP). This structured questionnaire for administration by self-report or assisted interview was co-produced with a working group of eight older people, aged over 65 years, many living with dementia, and their family carers, who collaborated with researchers to design and refine an adapted questionnaire based on ASCOT-SCT4 [27]. Unlike ASCOT-ER developed with adults with learning disabilities [16], ASCOT-ER OP does not include illustrations. These were found to distract, confuse or irritate respondents; instead, it relied on simplified text and layout to improve respondents’ ability to understand and respond to questions [27].

This study is the first to evaluate the measurement properties of ASCOT-ER OP, which is an important step in developing new or adapted measures. We apply exploratory factor analysis (EFA) combined with Rasch analysis using data collected from adults in the UK to establish ASCOT-ER OP’s structural validity against the ASCOT-SCT4, following a similar approach to psychometric studies of other adaptations designed to support inclusion e.g., ASCOT-ER [28]. Rasch analysis was also applied to determine if there were any issues with the rating scale or evidence of differential item functioning (DIF) by age group (18–64 years, over 65s), where ASCOT-ER OP is used to collect data with younger adults rather than over 65s (i.e., the target population it was developed for).

## Methods

### Data and sample

Data were collected by the British Red Cross (BRC), who were granted a not-for-profit license by University of Kent to use ASCOT-ER OP, and wished to integrate the measure into routine data collection within their Support at Home services (SAH) delivered across the UK. SAH offers one-to-one support for up to 12 weeks following illness or hospital admission to provide emotional support and practical assistance, build confidence, and refer or signpost to other organisations to meet people’s needs. It focussed on avoiding or delaying use of other services (e.g., residential care) and managing risks of immediate hospital re-admission. Core components included needs assessment, goal setting and support planning, welfare checks, community transport, practical and emotional support, and advice on how to navigate other services.

Data were collected by BRC staff as part of a SAH client’s initial assessment of need (baseline) and about three months later (follow-up). ASCOT-ER OP (https://research.kent.ac.uk/ascot/) was incorporated into a data collection Toolkit. The toolkit included written instructions for BRC staff on how to ask the questions and record responses. In particular, it was explained that the ASCOT-ER OP is a structured questionnaire designed to understand people’s views, so the questions should be asked as written, and answers recorded based on the client’s indicated response. Interviewers were instructed to maintain a non-judgemental approach and give client’s space and time to express their views, without influencing them. The questions should be asked as written, but flexibility applied to clarify questions (if asked). In addition, BRC staff completed a 20–30 min online e-learning Module and 20–30 min workshop exercise before implementation. The training explained the purpose and process of data collection from referral to final record, including ASCOT-ER OP. In addition, online drop-in sessions or submit queries by email were offered to all staff, post-implementation.

Within the toolkit, the eight ASCOT-ER OP questions were asked by BRC staff and rated according to the client’s response as: ideal state (best QoL), no needs, some needs or high-level needs (worst QoL). The ASCOT-ER OP *Dignity* item is designed for people accessing social care. It is not typically collected at baseline because it asks about the person’s experience of support from *paid staff*. In this service, however, it was asked at baseline, even though not all clients had contact with services. Data collected did not record whether or not the person already had support, so it was not possible to control for this. Therefore, we only used data collected between October 2023 and April 2025 at follow-up (*n* = 695), after SAH, at which point all clients had accessed at least one service (i.e., SAH itself).

Data collected by BRC were extracted, with advice from their Data Protection Officer, to prepare a dataset to share with researchers at the University of Kent that would be fully anonymised. The dataset included age group (10-year bands), sex (man/woman) and the ASCOT-ER OP SCRQoL rating for each domain. These data were securely transferred from BRC to University of Kent for analysis. Data were only accessible to the researcher, who conducted data analysis (SR).

Ethical review and approval was not sought on the basis that the study involved secondary analysis of anonymised data.

### Statistical analysis

The study’s aim was to evaluate the structural validity of ASCOT-ER OP [27], an easy read text adaptation of ASCOT-SCT4 designed for older adults [1]. Psychometric studies of ASCOT-SCT4 have found that the eight SCRQoL items load onto a single factor [1] and it has good construct validity, internal consistency and feasibility, across age groups and adults with different support needs [6, 7]. In this study, we applied exploratory factor analysis (EFA) to establish whether the ASCOT-ER OP, an adapted version of ASCOT-SCT4, retained the single factor structure of ASCOT-SCT4. Since some adaptations of the ASCOT-SCT4 have been found not to align to the original measure’s single factor structure (e.g. ASCOT-Proxy Proxy) [29], we apply EFA rather than confirmatory factor analysis. Rasch analysis was also applied to further evaluate measurement properties based on the factor structure identified by EFA [30, 31].

Rasch analysis was conducted in WINSTEPS 3.91.1 and other analyses in STATA 19.

#### Exploratory factor analysis

EFA was conducted to establish the structural properties and validity of ASCOT-ER OP. To confirm the data adequacy for factor analysis, the Kaiser–Meyer–Olkin (KMO) measure of sampling adequacy and Bartlett’s test of sphericity were conducted before proceeding to EFA. ASCOT measures apply a four-level rating scale for each QoL domain [5] and generate ordinal data. Therefore, we applied ordinal EFA using polychoric correlation matrices [32, 33]. Horn’s parallel analysis [34–36] guided the retention of factors. Eigenvalues were estimated through 5000 random correlation matrices using principal components analysis. The estimated Eigenvalues were then compared to observed Eigenvalues. Factors were only retained if observed exceeded estimated Eigenvalues, randomly [37, 38].

#### Rasch analysis

The combination of classical test theory (i.e. EFA) and item response theory (i.e., Rasch) in psychometric evaluation of measures is well-established and has been applied to other ASCOT measures (e.g., ASCOT-Proxy [9]). Rasch analysis generates a mathematical measurement model (the Rasch model) that is tested against empirical data. In this analysis, we applied the partial credit model because each ASCOT-ER OP item has a unique set of ordered options [39].

Model fit was considered and evaluated against established criteria. First, to evaluate the model assumption of unidimensionality [40], principal component analysis of the standardised residuals was conducted; Eigenvalues of < 2 to the first principal component of standardised residuals support the assumption of unidimensionality [41, 42]. Local independence, another assumption of the Rasch model [40], was assessed by examination of standardised residual correlations for pairwise combinations of items. Local independence was indicated by positive correlations of < 0.20 [43, 44]. To evaluate overall model fit, a standard deviation of < 1.50 for the item summary residual statistic was taken to be acceptable [45]. This criterion was preferred to the overall summary Chi-square interaction fit statistic, which can be sensitive to large sample sizes [46], as in this study. INFIT and OUTFIT mean square item statistics of 0.70–1.30, without adjustment for sample size, were taken to indicate good fit [47].

The following criteria were applied to evaluate the ordered category rating scale functionality: outfit mean square (OUTFIT MNSQ) of < 2; increased average measures and Rasch–Andrich thresholds between categories; and an increase of Rasch–Andrich category thresholds of 1.40–5.00 logits [48]. Since step calibration estimates can be imprecise when there are ≤ 10 cases per response category, we considered also the number of observations by response category [48].

ASCOT-ER OP was designed with and for older people, aged 65 or over, with mild cognitive impairment and mild-to-moderate dementia [27]. However, SAH included some adults, under 65 years. We retained the data collected from adults aged 18–64 in our analysis. The rationale was that there has been interest among users of ASCOT-ER OP, including BRC, in applying the measure across age groups. Psychometric evidence would be useful in guiding whether and how the measure is applied in this way. In Rasch analysis, specifically, we considered differential item functioning (DIF) by age group (18–64 years compared to 65 + years). DIF is an indicator of whether items function differently when completed by respondents from different subgroups. It looks at the different probability of rating an item by characteristic, after controlling for overall scale scores. In the analysis, age group of 18–64 years was used as the baseline group. The Mantel-Haenszel chi-square test was applied for pairwise comparisons, with a significance level of *p* < 0.006 (Bonferroni adjusted) indicative of DIF. A criterion of DIF contrast of ≥ 0.64 logits was used to identify moderate-to-large DIF, between groups [49].

## Results

Sample descriptive statistics are presented in Table 1. The sample were majority female (61.5%) and over 65 years (82.6%). Missing data for all ASCOT-ER OP items were less than six per cent. Low % missing values is consistent with other ASCOT measures [7, 29] and is taken as an indicator of feasibility of completion. Interestingly, the highest % missing data was for *Personal safety*, whereas it is typically *Dignity* due to its complexity. In discussing the results with BRC, it was noted that some BRC staff felt reticent to ask or probe about safety due to perceived sensitivity of the topic and their anxiety that it may evoke an emotional response. There was also feedback that some staff found it difficult to connect the question to the impact of SAH, despite the stated aims of SAH to improve confidence and independence, which can be related to clients’ anxiety about falling or other safety-related issues. These perceptions and the staff’s discomfort in asking the question may have affected its completion.

**Table 1:** Sample characteristics (*n* = 695)

	Frequency (%)
Sex	
Male	262 (37.7%)
Female	427 (61.4%)
Missing	6 (0.9%)
Age group	
Under 45 years	11 (1.9%)
45–54 years	26 (3.7%)
55–64 years	72 (10.4%)
65–74 years	123 (17.7%)
75–84 years	252 (36.3%)
85 or over	199 (28.6%)
Missing	10 (1.4%)
ASCOT-ER OP: missing data	
1. Control over daily life	9 (1.3%)
2. Personal comfort & cleanliness	8 (1.4%)
3. Food & drink	7 (1.0%)
4. Home comfort & cleanliness	11 (1.6%)
5. Social participation	25 (3.6%)
6. Personal safety	40 (5.8%)
7. Occupation	26 (3.7%)
9. Dignity	23 (3.3%)

### Exploratory factor analysis

The KMO measure of sampling adequacy and Bartlett’s test of sphericity were adequate for factor analysis (KMO = 0.84; χ^2^ (28) = 1658.56, *p* < 0.001). A single factor solution was supported by Horn’s parallel analysis. Observed Eigenvalues exceeded the random principal component Eigenvalue for the first factor only; the adjusted Eigenvalue for the second factor was less than one (0.98). All items loaded onto the single factor with factor loadings ≥ 0.40 [50] (see Table 2). High unique variance (≥ 0.60) was observed for *Personal safety* and *Dignity*. This is consistent with EFA conducted with data collected using other ASCOT measures that are adapted from ASCOT-SCT4 (e.g., ASCOT-Proxy [9]) and ASCOT-SCT4 itself [1].

**Table 2:** Exploratory factor analysis

	Factor loadings	Uniqueness
1. Control over daily life	0.74	0.45
2. Personal comfort & cleanliness	0.82	0.33
3. Food & drink	0.76	0.42
4. Home comfort & cleanliness	0.75	0.44
5. Social participation	0.71	0.49
6. Personal safety	0.63	**0.60**
7. Occupation	0.75	0.43
9. Dignity	0.46	**0.78**

Items with uniqueness ≥ 0.60 shown in bold

### Rasch analysis

Overall fit of observed data to the Rasch model was good. The standard deviation of the item summary residual statistic was 0.85 (i.e., below 1.5), which indicated acceptable fit. The overall Chi-square statistic was also insignificant (χ^2^ (7452 df) = 7312.94, *p* = 0.87). The Eigenvalue for the first principal component of residual was 1.89 (i.e., below the criterion of two), which supports the assumption of unidimensionality. Pairwise positive correlations of standardised residuals were found for *Personal comfort and cleanliness* with *Home comfort and cleanliness* (0.20) and *Social participation* with *Occupation* (0.17). The former is just above the standard critical value of 0.20, which *may* indicate violation of the assumption of local independence of items, but is inconclusive. Whilst the application of standard critical values is widespread and 0.20 is commonly applied, residual correlations of > 0.30 are *most likely* to indicate local dependence [44], so a borderline finding ought to be interpreted with caution.

INFIT and OUTFIT mean square values were all within the range of 0.7–1.30 [47] in the Rasch model, except the OUTFIT MNSQ for *Dignity* (1.88). However, this finding is not a particular cause for concern. Issues with outlier sensitive fit are less problematic than inlier sensitive fit issues. Since the OUTFIT MNSQ is between 1.30 and 2.0, it indicates an *overfit* to the Rasch model that is unproductive for measurement, but not degrading to the overall measure (Table 3).

**Table 3:** Item statistics

	Item difficulty	Standard error	INFIT MNSQ^a^	OUTFIT MNSQ^a^	Point-measure correlation
1. Control over daily life	0.27	0.07	0.94	0.97	0.71
2. Personal comfort & cleanliness	− 0.62	0.08	0.84	0.79	0.68
3. Food & drink	− 0.55	0.08	0.91	0.92	0.63
4. Home comfort & cleanliness	− 0.45	0.08	0.99	0.97	0.63
5. Social participation	1.10	0.07	0.98	1.00	0.73
6. Personal safety	1.01	0.07	1.20	1.25	0.67
7. Occupation	0.70	0.07	0.90	0.89	0.75
8. Dignity	− 1.46	0.09	1.25	1.88	0.36

^a^MNSQ = Mean square

In addition to overall and item fit, criteria were also applied to evaluate the rating scales (Table 4). First, OUTFIT MNSQ for each response category was less than two, except for *Dignity* at ‘no needs’ (OUTFIT MNSQ = 2.28). An OUTFIT MNSQ of less than two indicates that there is more unexplained than explained noise in the observations [48]. Since the Rasch model is a stochastic model, it assumes relatively uniform levels of randomness throughout the data. Where this is violated, especially where there are high levels of unexplained randomness or ‘noise’, it indicates that parts of the data do not contribute to useful measurement [48]. Sometimes, it can be resolved by identification and removal of problematic cases, where the rating scale is applied in an unexpected or inconsistent manner. Inspection of the data highlighted the unusual finding of *n* = 3 cases of *Dignity* rated as ‘no needs’, despite QoL ratings of ‘some needs’ or ‘high level needs’ across all other domains. A further n = 30 cases had at least one of the other domains rated as some or high-level needs, when *Dignity* was rated at no needs. Re-running the analysis to omit these n = 3 and n = 30 cases, respectively, did not improve model fit with regard to this diagnostic criterion.

Second, the average category measure and Rasch–Andrich category thresholds increased by response category for all eight items. This indicated that there was no evidence of disordered thresholds between rating categories. Third, we considered the step difficulty increase for Rasch–Andrich thresholds against the criteria of between 1.40 and 5.00 logits for optimal distinguishability. There were ≤ 10 ratings of high-level needs for four domains (*Dignity*,* Home* and *Personal comfort and cleanliness*,* Food and drink*), which may result in imprecise estimates [48]. Three of these (*Dignity*,* Home comfort and cleanliness*,* Food and drink*) had step difficulty increases between ‘high-level’ and ‘some needs’ that were under the minimum threshold of 1.40 logits [48]. There was evidence of suboptimal distinguishability also between ‘some needs’ and ‘no needs’ for six domains: *Personal comfort and cleanliness* (1.17 logit), *Social participation* (0.91 logit), *Personal safety* (0.75 logit), *Dignity* (0.28 logit), *Food and drink* (0.26 logit) and *Home comfort and cleanliness* (0.07 logit).

**Table 4:** Rating scale diagnostics

	Observed count	Observed average	OUTFIT MNSQ^a^	Rasch–Andrich threshold	Category measure
1. Control over daily life					
High-level	16	− 0.87	0.71	None	− 3.07
Some needs	102	0.48	1.13	− 2.12	− 1.04
No needs	311	1.51	0.92	− 0.46	1.37
Ideal state	257	3.21	0.97	2.58	3.99
2. Personal comfort & clean					
High-level	6	− 0.95	0.88	None	− 3.64
Some needs	45	0.01	1.11	− 1.74	− 1.81
No needs	262	0.93	0.56	− 0.57	0.31
Ideal state	374	2.86	0.86	2.31	2.83
3. Food and drink					
High-level	9	− 1.28	0.61	None	− 3.16
Some needs	35	0.03	0.92	− 1.16	− 1.62
No needs	252	0.99	0.93	− 0.90	0.16
Ideal state	392	2.68	0.98	2.06	2.65
4. Home comfort & clean					
High-level	9	− 0.48	1.22	None	− 3.15
Some needs	35	0.03	0.92	− 1.12	− 1.62
No needs	280	1.06	0.95	− 1.05	0.29
Ideal state	360	2.79	0.96	2.27	2.95
5. Social participation					
High-level	47	− 0.07	1.11	None	− 1.97
Some needs	120	0.65	1.00	− 1.75	− 0.21
No needs	316	1.88	0.92	− 0.84	2.03
Ideal state	187	3.52	0.98	2.58	4.80
6. Personal safety					
High-level	46	0.05	1.32	None	− 1.89
Some needs	107	0.89	1.32	− 1.55	− 0.19
No needs	291	1.83	1.20	− 0.80	1.87
Ideal state	211	3.23	1.20	2.35	4.49
7. Occupation					
High-level	22	− 0.34	0.94	None	− 2.83
Some needs	130	0.54	0.93	− 2.33	− 0.71
No needs	318	1.71	0.85	− 0.47	1.89
Ideal state	199	3.63	0.85	2.80	4.63
8. Dignity					
High-level	3	− 0.95	1.08	None	− 3.74
Some needs	14	− 0.25	1.04	− 0.83	− 2.25
No needs	119	1.06	2.28	− 0.55	− 0.85
Ideal state	536	2.03	1.20	1.38	1.12

^a^MNSQ = Mean square

Therefore, we explored whether distinguishability could be improved by collapsing categories together. Categories were collapsed for all instances of ≤ 10 ratings per category, i.e., data were combined for ‘high-level’ and ‘some needs’ for *Dignity*,* Home* and *Personal comfort and cleanliness*, and *Food and drink*. The findings from this alternative model are shown in Table 5. This model had acceptable fit with an item summary residual statistic of 0.61 (i.e., below 1.50), an insignificant overall Chi-square statistic (χ^2^ (7324 df) = 7189.51 *p* = 0.87) and item INFIT and OUTFIT mean square values between 0.70 and 1.30 [47], except for *Dignity* (OUTFIT MNSQ = 1.89). In reviewing the rating scale diagnostics (see Table 5), the step difficulty increases in Rasch–Andrich thresholds were within the 1.40–5.00 logit range, except for between ‘some needs’ and ‘no needs’ for *Personal safety* (0.77 logits) and *Social participation* (0.93 logits). We did not further explore whether collapsing categories could improve model fit, since it would move away from the ASCOT four-level rating categories that underpin all ASCOT measures [1]. Rather, suboptimal distinguishability at these thresholds is noted as a characteristic of the measure.

**Table 5:** Rating scale diagnostics (alternative model with collapsed categories)

	Observed count	Observed average	OUTFIT MNSQ^a^	Rasch–Andrich threshold	Category measure
1. Control over daily life					
High-level	16	− 1.26	0.76	None	− 3.46
Some needs	102	0.12	1.12	− 2.16	− 1.40
No needs	311	1.18	0.91	− 0.44	1.04
Ideal state	257	2.88	0.97	2.60	3.65
2. Personal comfort & clean					
High-level or some needs	51	− 0.47	1.09	None	− 2.60
No needs	262	0.60	0.56	− 1.40	− 0.05
Ideal state	374	2.52	0.86	1.40	2.49
3. Food and drink					
High-level or some needs	44	− 0.60	0.89	None	− 2.77
No needs	252	0.65	0.93	− 1.40	− 0.23
Ideal state	392	2.34	1.00	1.40	2.31
4. Home comfort & clean					
High-level or some needs	44	− 0.43	0.96	None	− 2.80
No needs	280	0.72	0.94	− 1.58	− 0.09
Ideal state	360	2.46	0.97	1.58	2.62
5. Social participation					
High-level	47	− 0.45	1.13	None	− 2.33
Some needs	120	0.30	0.99	− 1.76	− 0.56
No needs	316	1.55	0.92	− 0.83	1.70
Ideal state	187	3.19	0.98	2.59	4.47
6. Personal safety					
High-level	46	− 0.33	1.34	None	− 2.24
Some needs	107	0.54	1.31	− 1.56	− 0.53
No needs	291	1.50	1.21	− 0.79	1.54
Ideal state	211	3.09	1.19	2.35	4.16
7. Occupation					
High-level	22	− 0.77	0.95	None	− 3.20
Some needs	130	0.19	0.93	− 2.36	− 1.07
No needs	318	1.38	0.84	− 0.46	1.56
Ideal state	199	3.30	0.85	2.82	4.30
8. Dignity					
High-level or some needs	17	− 0.72	1.01	None	− 3.44
No needs	119	0.71	2.30	− 0.90	− 1.32
Ideal state	536	1.70	1.22	0.90	0.79

^a^MNSQ = Mean square

In addition to the overall model fit and rating scale diagnostics, we also considered differential item functioning (DIF) by age group. Significant Mantel-Haenszel chi-square tests (*p* < 0.006) were observed for the comparisons in *Personal safety* (χ^2^=8.33; *p* = 0.004) and *Home comfort and cleanliness* (χ^2^=10.05; *p* = 0.002). Both had DIF contrast values just under the criterion of ≥ 0.64 logits used to identify moderate-to-large DIF (− 0.63 and 0.62, respectively) [49]. Older people, aged 65 or over, were more likely to respond that they had their preferred QoL in *Home comfort and cleanliness* compared to 18- to 64-year-olds. In *Personal safety*, 18- to 64-year-olds were more likely to respond that they had their preferred QoL than older people.

## Discussion

This study evaluated the structural validity of ASCOT-ER OP using EFA and Rasch analysis. Using EFA, we found that the measure’s structure aligns with ASCOT-SCT4, i.e., a single factor structure [1]. In Rasch analysis, we found that ASCOT-ER OP had acceptable fit to the unidimensional Rasch model. Of the eight ASCOT-ER OP items, only *Dignity* was not a good fit to the model based on an OUTFIT mean square value of > 1.30; since it was also < 2.00, however, the overfit of *Dignity* to the Rasch model indicates that it does not optimally contribute to measurement but does not degrade the measure. On balance, the evidence does not indicate a serious issue with *Dignity*, even if not optimal, and it seems to be a characteristic shared with the original measure, ASCOT-SCT4 (see Online Appendix).

Whilst there was no evidence of disordered threshold, the analysis highlighted issues with the response categories for some items. An OUTFIT MNSQ of > 2.00 for *Dignity* at ‘no needs’ indicated that there was more unexplained than explained noise in the data. This finding was unexpected, especially since Rasch analysis of ASCOT-SCT4 data collected with community-dwelling older adults had acceptable OUTFIT MNSQ (see Online Appendix). Whilst the observed unpredictability of rating may be indicative of an issue with *Dignity*’s ‘no needs’ statement, it could be due to data collection characteristics. In discussing study findings with BRC, it was identified that some BRC staff had incorrectly assumed that the item was ‘not applicable’ if the only service the client received was SAH. In that case, they had rated ‘no needs’, rather than asking the question of the client. These responses of ‘no needs’ were combined with those who had asked *Dignity* to establish the impact of SAH and/or other services. Unfortunately, the dataset did not record whether other services were accessed after SAH, or not, so we cannot explore further with this dataset. It would usefully be explored in future studies. This also highlights the importance of interviewer instructions; although the written instructions and training explained that all questions should be asked, we recommended that the information be amended to explicitly say that the Dignity question should be asked of clients, even if SAH is the only service they are accessing.

Rasch analysis also highlighted issues with response categories. Rasch analysis, after collapsing categories to avoid imprecise estimates due to ≤ 10 cases per category, highlighted persistent suboptimal distinguishability at ‘some needs’ to ‘no needs’ for *Social participation* and *Personal safety.* The finding at this threshold for *Social participation* replicates a known issue that has been found for ASCOT-SCT4 (Online Appendix) and ASCOT-Proxy completed by care home staff [9]. The wording of response options could be further explored to establish whether adjustment could improve the psychometric properties; however, we note that the response statements have already been carefully developed and refined using qualitative evidence for ASCOT-SCT4 [1] and its adaptations, including ASCOT-ER OP [27], it may be that the issue is not resolvable through further edits within the parameters of the ASCOT four-level response definitions. Also, because ASCOT-SCT4 was developed to cover domains of importance to people accessing social care and has been widely used since 2012 [5], it may not be feasible to retrospectively recommend item omission due to suboptimal distinguishability, whether for ASCOT-SCT4 or adapted measures, especially as affected domains vary slightly between data collections. Instead, we note it as a characteristic of data collected using ASCOT-SCT4 and its adaptations.

Rasch analysis also highlighted differential item functioning (DIF) by age group. Respondents aged 18-to-64 and 65 + years had different conceptualisations of *Personal safety* and *Home comfort and cleanliness*. Qualitative literature supports that older adults’ understanding of safety can differ from adults, aged 18–64 [16], and that ageing can influence the experience of place attachment and meanings associated with ‘home’ [51–53]. Because the measure was specifically developed with older adults [27], we would recommend that it is used to collected data from older adults only. If it is used to collect data from adults aged 18–64 years and over 65s, we recommend the separate reporting of data by age group. Further investigation of DIF in future studies is warranted.

This study has some limitations. First, while psychometric studies can be conducted on routine data (e.g [9]). , and it offers insights into how measures work in this context, which is an area of interest in policy and practice [13, 54, 55], it also presents potential limitations: for example, non-compliance of staff to the written instructions and training in how data are to be collected. Therefore, it is desirable to replicate analyses on data collected under a scientific protocol in future studies. Second, as the data analysed in this study were from routine administrative data collected by BRC, we were not able to conduct construct validity analyses due to the lack of suitable data in the dataset (e.g., measures of similar constructs) for convergent validity by hypothesis testing. Likewise, there remain gaps in psychometric evidence for the measure’s test-retest reliability and responsiveness, which would be usefully addressed in future studies. Third, we note that the study sample was adults accessing a particular community-based service, SAH. Future studies may wish to consider how the measure performs in other contexts and settings (e.g., homecare). Finally, the study that developed ASCOT-ER OP was underpinned by co-production principles and involved co-design with a working group of older adults [27]. This psychometric analysis was conducted outside of the main study, which precluded the involvement of the working group. In retrospect, it would have been ideal and more aligned with the inclusive and participatory co-production ethos of the project to have integrated the psychometric study into the overall project.

## Conclusions

The ASCOT-ER OP is a co-designed measure informed by cognitive interviews that offers a way of capturing self-report from older adults, who may be currently underserved by standard questionnaires. This first psychometric (structural validity) study of the ASCOT-ER OP used secondary data that was collected by British Red Cross in individual needs assessment and evaluation as part of Support at Home services in the UK. In EFA, ASCOT-ER OP was found to have a single factor structure, which is consistent with the ASCOT-SCT4 from which ASCOT-ER OP was adapted. Rasch analysis had acceptable model fit overall, which further supported the measure’s validity. Based on evidence of DIF by age group (18–64 and 65 + years), we recommend that ASCOT-ER OP is either used only with the target population for which it was developed (i.e., older adults) or that data are reported separately by age group. The study provides preliminary evidence of the measure’s structural validity, which would usefully be replicated using data collected in future research studies under scientific protocol, and supports its use to support more inclusive data collection in adult social care research and evaluation.

## Supplementary Information

Below is the link to the electronic supplementary material.

Supplementary Material 1

## Data Availability

The anonymised secondary data analysed during the current study are not publicly available, because they were shared between British Red Cross and the research team at the University of Kent. No further sharing permission was granted, due to data sensitivity and to further mitigate against re-identification risk.
