# Supplementary material for: The adult social care outcomes toolkit easy read for older people (ASCOT-ER OP): an exploratory factor analysis and Rasch validation study
Source: Qual Life Res. 2026 Mar 13;35(4):95. doi: 10.1007/s11136-026-04169-0 (PMC12987906; doi:10.1007/s11136-026-04169-0)
Supplement: Supplementary file 1 — Supplementary Material 1 [file 11136_2026_4169_MOESM1_ESM.docx]

**Appendix: Rasch analysis of adult social care survey (ASCS) data**

**METHODS**

The adult social care survey (ASCS) is a national survey of adults accessing publicly managed social care in England.^[[1]](#footnote-1)^ This analysis drew on the 2023/24 ASCS data collected from older adults, aged 65 or over, accessing publicly managed community-based services in England, who completed a standard version of ASCOT-SCT4, which is routinely included in the ASCS.

We conducted a Rasch analysis of ASCOT-SCT4 data to allow comparison of the results against the ASCOT-ER OP. Whilst studies have conducted EFA on ASCOT-SCT4 data (e.g. Netten et al, 2012) or Rasch analysis with other subsamples of the ASCS data (e.g., Rand et al, 2024), no prior studies have explored the Rasch model with ASCOT-SCT4 data for community-dwelling older adults, who access adult social care services (e.g., home care).

This subsample (n=23,107) was majority female (66.1%) and had % missing data for the ASCOT-SCT4 items of less than six per cent across items. Items with highest per cent missing data were *Dignity* (5.8%) and *Occupation* (4.6%); other items had between two and four per cent missing data.

The aim of the analysis presented here is to allow comparison with the findings of the Rasch analysis with the ASCOT-ER OP data and, specifically, to identify how far issues with the new measure correspond to the properties of the original measure (i.e., ASCOT-SCT4) and where they diverge. This will aid decision-making on further refinement of the ASCOT-ER OP.

The Rasch analysis followed the data analysis procedure outlined in the main manuscript.

**RESULTS**

The observed data from the ASCS had good fit to the Rasch model based on an item summary residual statistic of 0.87 (i.e., below the cut-off of 1.50 and close to a value of 1.00, which reflects the ‘perfect’ fit) and Chi-square statistic that was not significant (χ² (277,741 df) = 274,741.19, p=1.000). In addition, Rasch model assumptions of unidimensionality and local independence of items were not violated (i.e., the Eigenvalue for the first principal component of residual was below the cut-off value of two (1.54) and no positive correlations of the standardised residuals were observed for pairwise comparisons between items). INFIT and OUTFIT mean square values (Table I) were also all within the range of 0.70 to 1.30, which indicates acceptable fit to the model [30], except for the OUTFIT mean square for *Dignity* (1.36).

Table I. Item statistics

|  | **Item difficulty** | **Standard Error** | **INFIT**  **MNSQ¹** | **OUTFIT MNSQ¹** | **Point-measure correlation** |
| --- | --- | --- | --- | --- | --- |
| 1. Control over daily life | 0.91 | 0.01 | 0.95 | 0.95 | 0.71 |
| 2. Personal comfort & cleanliness | -0.53 | 0.01 | 0.91 | 0.91 | 0.64 |
| 3. Food & drink | -0.69 | 0.01 | 0.98 | 0.97 | 0.58 |
| 4. Home comfort & cleanliness | -0.91 | 0.01 | 1.02 | 1.03 | 0.57 |
| 5. Social participation | -0.60 | 0.01 | 1.07 | 1.15 | 0.54 |
| 6. Personal safety | 0.82 | 0.01 | 0.95 | 0.93 | 0.71 |
| 7. Occupation | 1.52 | 0.01 | 0.91 | 0.90 | 0.75 |
| 8. Dignity | -0.52 | 0.01 | 1.18 | 1.36 | 0.53 |

¹ MNSQ = Mean square

The OUTFIT MNSQ was less than two across all response categories for the eight ASCOT-SCT4 items (see Table II). Also, there was a step increase in the Rasch-Andrich threshold and category measures between response options for all ASCOT-SCT4 items, except for *Social participation.* At this threshold, there was a minimal step decrease in Rasch-Andrich values (0.07 logit), which indicated suboptimal distinguishability between these response categories. Likewise, there was evidence of less-than-optimal distinguishability between ‘some’ and ‘no needs’ for *Personal comfort and cleanliness* (0.72 logits), *Food and drink* (0.86 logits) and *Home comfort and cleanliness* (1.24 logits).

None of the categories had ratings by less than 10 people, although high-level needs were selected by a relatively low % of the sample. This is a known characteristic of ASCOT data collections and is intentionally part of the design of ASCOT measures. ASCOT considers the theoretical state of unmet care needs (with potential or actual health consequences), even if this particular outcome state (and associated response option) is relatively uncommon under long-term care systems, where there is a statutory duty to identify and address such eligible unmet needs. Even if low % of samples select high-level needs, it is theoretically important to retain the high-level needs response option, especially if the data are being used to assess individual care needs and/or evaluate care delivery and/or assess LTC system performance (for further discussion see Rand et al, 2024; Rand et al, 2025).

Table II. Rating scale diagnostics

|  | **Observed count** | **Observed average** | **OUTFIT**  **MNSQ¹** | **Rasch-Andrich threshold** | **Category measure** |
| --- | --- | --- | --- | --- | --- |
| **1. Control over daily life** |  |  |  |  |  |
| High-level | 1,572 | -0.07 | 1.18 | NONE | -2.19 |
| Some needs | 5,288 | 0.57 | 0.81 | -1.87 | -0.20 |
| No needs | 9,576 | 1.80 | 0.84 | -0.26 | 1.89 |
| Ideal state | 6,092 | 2.89 | 0.99 | 2.13 | 4.20 |
| **2. Personal comfort & clean** |  |  |  |  |  |
| High-level | 286 | -1.06 | 1.00 | NONE | -3.46 |
| Some needs | 1,412 | -0.18 | 0.80 | -1.57 | -1.77 |
| No needs | 10,309 | 1.15 | 0.91 | -0.85 | 0.33 |
| Ideal state | 10,595 | 2.45 | 0.95 | 2.42 | 3.02 |
| **3. Food and drink** |  |  |  |  |  |
| High-level | 323 | -1.00 | 1.16 | NONE | -3.33 |
| Some needs | 1,415 | -0.10 | 0.92 | -1.31 | -1.63 |
| No needs | 7,148 | 1.01 | 0.93 | -0.45 | 0.08 |
| Ideal state | 13,370 | 2.22 | 1.01 | 1.76 | 2.25 |
| **4. Home comfort & clean** |  |  |  |  |  |
| High-level | 187 | -0.97 | 1.24 | NONE | -3.88 |
| Some needs | 1,289 | -0.15 | 0.98 | -1.69 | -2.02 |
| No needs | 7,965 | 1.03 | 1.03 | -0.45 | 0.01 |
| Ideal state | 12.939 | 2.22 | 1.03 | 2.15 | 2.39 |
| **5. Social participation** |  |  |  |  |  |
| High-level | 457 | -0.57 | 1.70 | NONE | -2.88 |
| Some needs | 1,065 | .-0.07 | 1.04 | -0.74 | -1.46 |
| No needs | 6,930 | 1.00 | 1.16 | -0.81 | -0.01 |
| Ideal state | 13.944 | 2.15 | 1.04 | 1.56 | 2.12 |
| **6. Personal safety** |  |  |  |  |  |
| High-level | 1,846 | -0.22 | 0.98 | NONE | -1.96 |
| Some needs | 4,964 | 0.67 | 0.85 | -1.53 | -0.07 |
| No needs | 7,974 | 1.69 | 0.84 | -0.10 | 1.67 |
| Ideal state | 7,563 | 2.76 | 1.02 | 1.64 | 3.67 |
| **7. Occupation** |  |  |  |  |  |
| High-level | 2,811 | 0.10 | 1.02 | NONE | -1.60 |
| Some needs | 7,464 | 0.93 | 0.78 | -1.92 | 0.55 |
| No needs | 7,054 | 2.17 | 0.77 | 0.09 | 2.53 |
| Ideal state | 4,714 | 3.11 | 1.02 | 1.83 | 4.56 |
| **8. Dignity** |  |  |  |  |  |
| High-level | 302 | -0.62 | 1.46 | NONE | -3.63 |
| Some needs | 2,399 | 0.17 | 1.04 | -1.93 | -1.50 |
| No needs | 7,239 | 1.38 | 1.72 | 0.06 | 0.50 |
| Ideal state | 11,835 | 2.15 | 1.22 | 1.87 | 2.56 |

¹ MNSQ = Mean square

1. The ASCS data are publicly available online: NHS Digital, ‘Personal Social Services Adult Social Care Survey, England, 2023-24’ <https://digital.nhs.uk/data-and-information/publications/statistical/personal-social-services-adult-social-care-survey/england-2023-24> <Accessed 27 June 2025> [↑](#footnote-ref-1)
